# Replanted Teeth

**Published:** 1867-11

**Authors:** 


### REPLANTED TEETH.

On the 7th of July last, Mr. A. C., aged 20, called at my office to consult me in reference to an accident by which his upper central incisors and the right lateral had been knocked out. The accident occurred at 4½ P. M., and it was now 7½. He had in his pocket the centrals, attached together by a portion of the gum—the "*ligamentum dentis.*" The teeth, and the soft parts adherent to them, appeared perfectly dry. The hemorrhage was still active.

On examination, I found the socket of the right central badly fractured, and the soft parts about it much bruised and lacerated. The patient was pale, having a cadaverous look, a strumous appearance, and a despondent countenance. I have found since that his vital powers are not good.

I washed out the sockets with tepid water, and replaced the centrals, retaining them by a temporary compress, and sent him in search of the missing lateral. The distance was about two miles, and he was gone nearly two hours. At 9½ P. M., five hours after the accident, the lateral was replaced, after carefully washing out the socket with tepid water, and a gutta percha splint or compress was so adjusted as to hold them all in proper position. This splint was worn till 10 A. M. next day, when the teeth were found to be slightly adherent. As a precaution, the splint was replaced at night for a short time, and then wholly laid aside. Inflammation was combatted, locally and generally, by ordinary means, and really gave but little trouble.

This is the case I reported at the late meeting of the American Dental Association. I was requested by several to report progress again, and promised to do so, unless I lost the run of the case. The tooth in the fractured socket was the most difficult to manage. Like the rest, however, it became perfectly tight—firmer than before the accident. All progressed satisfactorily till about the middle of November, when a small alveolar abscess made its appearance over the right central, that with the fractured socket, giving the patient but little pain, and now causing no trouble. As soon as the patient can control his time, I expect to treat this. The other two teeth are not changed in color.

